# Toxic Effects of Polystyrene Microplastics and Sulfamethoxazole on Early Neurodevelopment in Embryo–Larval Zebrafish (*Danio rerio*)

**DOI:** 10.3390/toxics14010074

**Published:** 2026-01-14

**Authors:** Fantao Meng, Shibo Ma, Yajun Wang, Chunmei Wang, Ruoming Li, Jiting Wang

**Affiliations:** Key Laboratory of Efficient Utilization of Non-Grain Feed Resources (Co-Construction by Ministry and Province), Ministry of Agriculture and Rural Affairs, Shandong Provincial Key Laboratory of Animal Nutrition and Efficient Feeding, Department of Animal Science, Shandong Agricultural University, Taian 271017, China; mengfantao361@163.com (F.M.);

**Keywords:** polystyrene microplastics, sulfamethoxazole, embryotoxicity, integrated biomarker response

## Abstract

Microplastics (MPs) and antibiotics have emerged as contaminants of global concern, posing potential threats to ecosystem security and organismal health. To investigate the individual and combined toxicity of microplastics (PS-MPs) and sulfamethoxazole (SMX), we conducted a 120 h acute exposure experiment using embryo–larval zebrafish as a toxicological model. Our findings demonstrate that both PS-MPs and SMX can induce neurodevelopmental toxicity in embryo–larval zebrafish during embryonic development. Notably, PS-MPs and SMX exerted a significant synergistic effect. PS-MPs 1 µm in diameter were restricted to the chorion surface of pre-hatching zebrafish, whereas post-hatching, PS-MPs accumulated mainly in the gut and gills, with accumulation levels increasing progressively with exposure duration. Individual exposure to PS-MPs or SMX reduced spontaneous locomotion, decreased heart rate, and shortened body length in embryo–larval zebrafish. In addition to exacerbating these effects, coexposure further increased the incidence of malformations such as pericardial effusion and spinal curvature. PS-MPs and SMX significantly decreased the levels of dopamine (DA), serotonin (5-HT), and γ-aminobutyric acid (GABA) in zebrafish while also suppressing acetylcholinesterase (AChE) activity and increasing acetylcholine (ACh) levels. Moreover, upon coexposure at high concentrations, PS-MPs and SMX acted synergistically to reduce the levels of DA and GABA. The downregulation of key neurodevelopmental genes (*elavl3*, *gap43*, and *syn2a*) and related neurotransmitter pathway genes indicates that PS-MPs and SMX impaired structural development and functional regulation of the nervous system. An integrated biomarker response (IBR) index confirmed that PS-MPs and SMX significantly enhanced developmental neurotoxicity during early neurodevelopment in embryo–larval zebrafish through synergistic effects. Our study provides critical toxicological evidence for the scientific assessment of the ecological risks posed by microplastic–antibiotic cocontamination.

## 1. Introduction

The widespread occurrence of microplastics (MPs) and antibiotics in aquatic systems has raised significant concern regarding their ecological risks. Notably, MP pollution has become pervasive across diverse aquatic environments, influenced by the characteristics of pollutants and the inflow of pollution sources, with particularly pronounced effects on the security of freshwater ecosystems [1]. MPs are defined as plastic particles with a diameter of less than 5 mm and exhibit diverse morphologies, such as fragments, films, and microbeads [2]. Annual global plastic production is immense; however, only approximately 9% of plastic waste is effectively recycled, with the vast majority ultimately entering the environment [3]. MPs have toxic effects on aquatic organisms via multiple primary pathways: (i) direct ingestion leading to physical injury and physiological impairment [3]; (ii) acting as vectors that adsorb and transport environmental pollutants (e.g., antibiotics), leveraging their surface hydrophobicity and high surface area to enhance combined toxicity [4], and (iii) leaching of additives used in MPs production (e.g., polybrominated diphenyl ethers, bisphenol A), leading to effects such as endocrine disruption and genotoxicity [5,6]. Previous studies have reported that MP concentrations in seawater range from approximately 3 to 23 µg/L, while in heavily polluted areas they can reach up to 4.5 mg/L [7,8]. In freshwater systems, MP abundance also varies substantially, from about 1 items m^−3^ in lightly polluted waters to as high as 10^6^ items m^−3^ in severely contaminated sites [9].

Antibiotics have been widely detected in aquatic environments because of their extensive use. Sulfonamide antibiotics, in particular, are frequently detected in global surface waters, with reported occurrence rates ranging from 50 to 80 China, their contamination levels show distinct spatial variability. The concentrations are relatively high in water resources such as the Huangpu River (259.6 ng/L) and Baiyangdian Lake (240 ng/L), while lower concentrations are found in the Yellow River (44 ng/L) and the Liao River (sulfamethoxazole, SMX, 60.9 ng/L) [10]. These concentrations are comparable to those reported in rivers worldwide, including the Rhine River in Germany (93 ng/L), the Thal River in Spain (67 ng/L), and several rivers in India (56.4–64 ng/L) [11,12,13].

The interactions between antibiotics and other pollutants, such as MPs, are key indicators for assessing the ecological risks of antibiotics. Antibiotics can bind to microplastics primarily through adsorption, partitioning and other processes [14]. Owing to their powerful sorption capacity, MPs can concentrate antibiotics at levels several orders of magnitude higher than those in water [15]. However, the resulting effects on biological toxicity are not uniform, depending on the properties of the MPs or the exposure duration. Liu et al. [16] demonstrated that MPs acted as carriers for tetracycline, significantly exacerbating the resulting intestinal and gill damage in grass carp. In contrast, Yu et al. [17] reported that MPs alleviated oxytetracycline-induced intestinal injury in zebrafish.

The natural environment is composed of a complex mixture of substances, and understanding the interactions between different pollutants is critical for developing effective strategies for aquatic ecosystem remediation. The combined effects of coexisting pollutants manifest as both physical adsorption and biological toxicity. In terms of physical adsorption, the binding of antibiotics to minerals, such as the adsorption of quinolones onto Fe-bearing minerals, can be either inhibited or enhanced by the presence of coexisting ions [18]. At the toxicity level, pollutants may act synergistically to amplify biological damage. For example, compared with individual exposure, coexposure of embryonic zebrafish to antibiotics and heavy metals induces more severe oxidative stress [19]. Therefore, elucidating the interaction mechanisms between specific pollutants is of fundamental importance. In this study, embryo–larval zebrafish were used as the experimental model. Embryo–larval zebrafish were exposed to polystyrene microplastics (PS-MPs) and varying concentrations of SMX starting at 2 h postfertilization, with the goal of assessing the individual and combined toxic effects of these pollutants during early development. By systematically monitoring developmental endpoints, including hatchability, mortality, malformation rate, behavioural alterations, and cardiotoxicity, and integrating these data with analyses of neurotransmitter levels and neurodevelopment-related gene expression, we ultimately applied the integrated biomarker response (IBR) index to comprehensively evaluate the developmental neurotoxicity and interaction mechanisms of PS-MPs and SMX.

## 2. Materials and Methods

### 2.1. Materials

Adult wild-type zebrafish (AB strain) were obtained from a local commercial supplier. Broodstock were randomly selected regardless of sex, with an average body length of 35.0 ± 5.0 mm and a body weight of approximately 0.3 g. All zebrafish were acclimated under standard laboratory conditions for at least one month, with a photoperiod of 14 h light and 10 h dark, and fed three times daily. Males and females were transferred into the hatcheries at a ratio of 2:1. Spawning behaviour was triggered by light stimulation. The fertilized eggs were collected, rinsed thoroughly, and subsequently used for exposure, which commenced within 2 h postfertilization and lasted up to 120 h.

### 2.2. PS-MPs and SMX Exposure of Zebrafish Embryos

For the embryotoxicity assay, monodisperse PS-MPs with a diameter of approximately 1 µm were used. To visualize the in vivo distribution of PS-MP, monodisperse red fluorescent PS-MPs of equivalent diameter (excitation wavelength, 620 nm; emission wavelength, 680 nm) were employed (Figure S1). Both types of PS-MPs were obtained from Tianjin BaseLine ChromTech Research Centre (Tianjin, China). Six groups were established for PS-MPs and SMX (Macklin, Shanghai, China) exposure of embryonic zebrafish, which were named CK (control check), 10 SMX (10 µg/L SMX), 100 SMX (100 µg/L SMX), Ps (500 µg/L PS-MPs), Ps + 10 SMX (500 µg/L PS-MPs + 10 µg/L SMX) and Ps + 100 SMX (500 µg/L PS-MPs + 100 µg/L SMX), with five replications for each group (*n* = 250/each, 5 replicates of 50 zebrafish). The 50 × 10^3^ solution was diluted fifty-fold and used to prepare the exposure solutions of PS-MPs and SMX. The medium consisted of a 50 × E3 stock solution (NaCl 14.61g, KCl 0.6337 g, CaCl_2_ 1.8315 g, MgSO_4_·7H_2_O 3.697 g) in purified water, with pH 7.0 ± 0.5, conductivity 200 ± 10 µS/cm, dissolved oxygen 7.0 ± 0.5 mg/L and temperature 28.0 ± 1.0 °C. After preparation, the solution was thoroughly mixed for 5 min using an ultrasonic cleaner (Ningbo Scientz Biotechnology Co., Ltd., Ningbo, China) operating at 100 W and 40 kHz. The exposure medium was replaced every 24 h. At the end of the 120-h exposure, the zebrafish were rinsed with deionized water, and samples from each treatment group were separately transferred into 1.5 mL cryovials and stored at −80 °C for subsequent analysis.

### 2.3. Analysis of PS-MPs Distribution in Zebrafish Embryos

One-micrometre monodisperse red fluorescent polystyrene microspheres of equivalent diameter were used to investigate the in vivo distribution of microplastics in embryo–larval zebrafish. Healthy embryos were randomly distributed into 6-well cell culture plates, with 50 embryos per well. Exposure commenced at 2 h postfertilization, with a consistent PS-MPs concentration of 500 µg/L in all treatment groups. The incubation conditions were maintained at 28.0 ± 0.5 °C, and the exposure medium was replaced every 24 h. During the exposure period, zebrafish embryos were sampled at 12 h intervals. Embryos were transferred to a culture dish and gently rinsed three times with 5 mL of deionized water using a pipette. This washing step was performed to remove residual exposure medium and any loosely attached or non-adhered MPs from the embryonic surface. Randomly selected embryos were anesthetized with 0.03% MS-222 (Sigma-Aldrich, Saint Louis, MO, USA) and observed under a fluorescence microscope (RIDET, Shanghai, China) for imaging (excitation wavelength, 620 nm; emission wavelength, 680 nm). The fluorescence intensity of the PS-MPs in the embryos was quantified using ImageJ-win64 (v1.54r, NIH, USA) software.

### 2.4. Detection of Basic Developmental Indicators

When the culture medium was changed, the egg membrane was removed, the dead embryo/larvae zebrafish were aspirated, and the embryo survival rate and hatching rate were calculated as follows: embryo survival rate (ESR, %) = embryo survival count/initial embryo number × 100% and hatching rate (%) = hatching number/initial embryo number × 100%.

During the exposure period from 2 to 120 hpf, malformed embryos (pericardial effusion, yolk sac edema, and spinal curvature) were identified and counted under a stereomicroscope (ZEISS, Shanghai, China) as follows: embryo malformation rate (EMR, %) = malformation count/hatching number × 100%.

Embryo–larval zebrafish were placed under a stereomicroscope and recorded using a ZEISS ZEN 3.2 (blue edition) imaging system. The following endpoints were quantified: spontaneous movements over 1 min at 24 hpf, heart rate over 30 s at 48 hpf, and body length at 120 hpf.

### 2.5. Neurotransmitter Level and Enzyme Activity Measurement

Larval zebrafish were accurately weighed and homogenized on ice in normal saline at a tissue-to-solution ratio of 1:9 (*w*/*v*). The homogenate was centrifuged at 4 °C and 3000 rpm for 20 min, and the supernatant was collected for subsequent assays. The levels of dopamine (DA), serotonin (5-HT), and γ-aminobutyric acid (GABA) in larval zebrafish were measured using ELISA kits (Jiangsu Meimian Industrial Co., Ltd., Yancheng, China). The acetylcholine (ACh) content, acetylcholinesterase (AChE) activity, and total protein (TP) concentration were determined with commercial kits (Nanjing Jiancheng Bioengineering Institute, Nanjing, China) following the manufacturers’ instructions (Text S1).

### 2.6. Quantitative Real-Time PCR

TRIzol (Invitrogen, Carlsbad, CA, USA) was used to extract total RNA, and the concentration of extracted RNA was detected using a nucleic acid protein detector (Denovix, Wilmington, NC, USA). cDNA was synthesized by reverse transcription following the manufacturer’s instructions for the reverse transcription kit (Aikori, Changsha, China).

The primers were designed using Primer 6.0 software on the basis of zebrafish gene sequences obtained from the NCBI database (https://www.ncbi.nlm.nih.gov, accessed on 20 April 2025). *β-actin* was used as the reference gene (Table 1), and all primers were synthesized by Beijing Tsingke Biotechnology Co., Ltd., Beijing, China. The relative expression of target genes was measured by quantitative real-time PCR.

### 2.7. Integrated Biomarker Response Index

The concept of the integrated biomarker response (IBR) index was first introduced by Beliaeff and Burgot to assess the toxicity of complex mixtures [20]. Sanchez subsequently modified the original IBR calculation method and proposed the second-generation integrated biomarker response index [21,22]. The calculation formula is as follows:

Y_i_ = log(X_i_/X_0_), Z_i_ = (Y_i_ − µ)/σ, A = Z_i_ − Z_0_, IBR = ∑ |A|.

where Y_i_ is the mean value of biomarkers under a specific treatment condition, X_0_ is the mean value in the control group, X_i_ is the standardized value, µ is the overall mean across all treatment conditions, σ is the overall standard deviation across all treatment conditions, Z_i_ is the normalized value, Z_0_ is the normalized value of the corresponding biomarker in the control group, and A represents the deviation index for each indicator. The sum of |A| yields the IBR value, which reflects the magnitude of the overall toxicity.

### 2.8. Statistical Analysis

The data were tested for normality using the Shapiro–Wilk test and for homogeneity of variance using Levene’s test and are presented as the mean ± SEM. The relative expression of target genes was calculated using the 2^−ΔΔCt^ method [23]. Statistical analysis was performed by one-way ANOVA followed by Tukey’s post hoc test with SPSS 22.0, and the difference was considered significant at *p* < 0.05. Figures were performed in GraphPad Prism (Version 8.0.2; GraphPad Software, Inc., San Diego, CA, USA).

## 3. Results

### 3.1. Distribution of PS-MPs in Zebrafish Embryos

**Figure 1:**
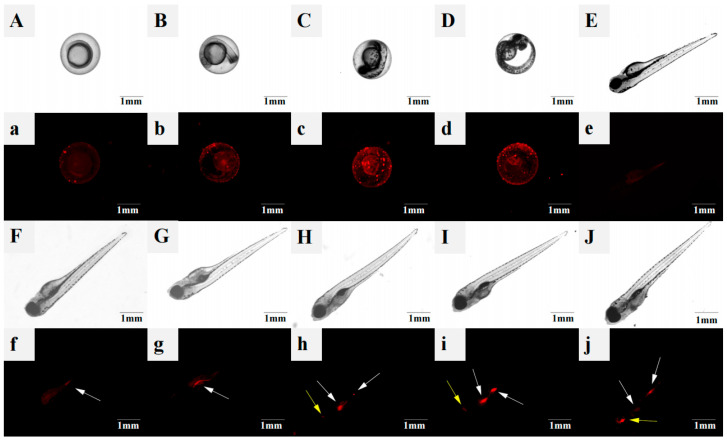
Absorption and accumulation of 500 µg/L PS-MPs in embryo zebrafish. bright-field, (**A**) 12 hpf, (**B**) 24 hpf, (**C**) 36 hpf, (**D**) 48 hpf, (**E**) 60 hpf, (**F**) 72 hpf, (**G**) 84 hpf, (**H**) 96 hpf, (**I**) 108 hpf, (**J**) 120 hpf. Fluorescent, (**a**) 12 hpf, (**b**) 24 hpf, (**c**) 36 hpf, (**d**) 48 hpf, (**e**) 60 hpf, (**f**) 72 hpf, (**g**) 84 hpf, (**h**) 96 hpf, (**i**) 108 hpf, (**j**) 120 hpf. White arrows denote intestinal accumulation and yellow arrows indicate gill accumulation.

**Figure 2:**
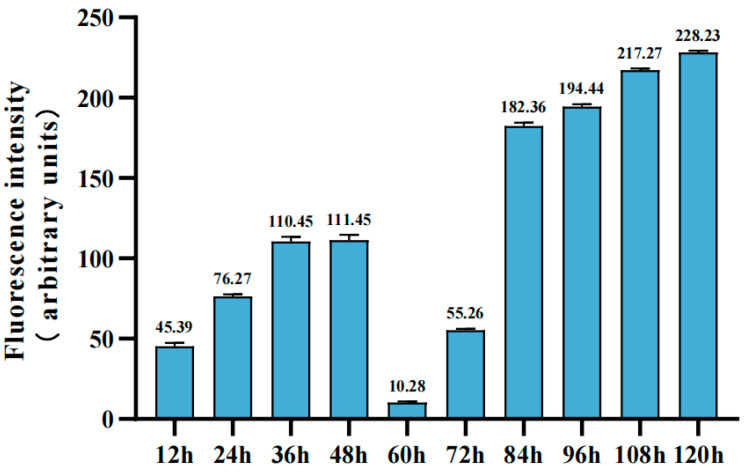
Comparison of relative fluorescence intensity of red fluorescent PS-MPs in embryo–larval zebrafish.

To investigate the accumulation of 1 µm PS-MPs in zebrafish embryos, their distribution was visualized using fluorescence microscopy (Figure 1). The chorion acted as a barrier against 1 µm PS-MPs during prehatching (Figure 1A–D). The particles adhered only to the chorion surface, with no fluorescent signal detected inside the embryos. Moreover, the adhesion of PS-MPs to the chorion increased with prolonged exposure. PS-MPs (1 µm) were predominantly accumulated in the intestine and gills posthatching (Figure 1F–J). At 72 hpf, PS-MPs were first observed in the intestine (Figure 1F), and by 108 hpf, apparent accumulation was also evident in the gills (Figure 1I). During the larval stage, PS-MPs accumulation increased progressively with increasing exposure (Figure 2).

### 3.2. Effects of PS-MPs and SMX on the Growth and Development of Embryo–Larval Zebrafish

#### 3.2.1. Effects on the Spontaneous Movement of Zebrafish Embryos

**Figure 3:**
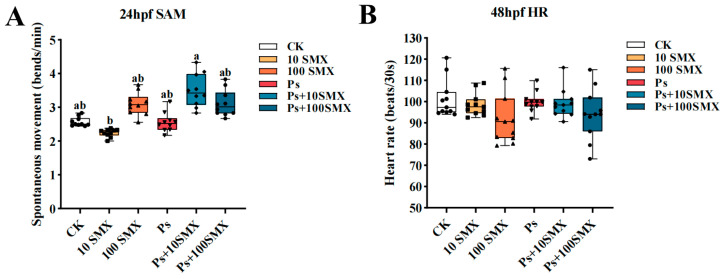
Effects of PS-MPs and SMX exposure on basic developmental indicators in embryo–larval zebrafish. (**A**) Spontaneous movement counts at 24 hpf; (**B**) Heart rate at 48 hpf. Note: *n* = 10 replicates (15 larvae each), totaling 150 larvae per group (Different lowercase letters refer to differences at significance level *p* < 0.05 among different exposure groups).

Spontaneous movement is driven by rhythmic electrical signals generated by the central nervous system of the embryo, representing a direct manifestation of the initial establishment of neural and muscular function and serving as a primary indicator for detecting early neurotoxicity. As shown in Figure 3A, at 24 hpf, the spontaneous movement frequency clearly decreased in the 10 µg/L SMX exposure group compared with that in the CK group, although the difference was not statistically significant (*p* > 0.05). The spontaneous movement frequency was significantly higher in the Ps + 10 µg/L SMX coexposure group than in the 10 µg/L SMX exposure group (*p* < 0.01). The remaining exposure groups tended towards increased movement, but none of these changes reached statistical significance compared with those in the CK group (*p* > 0.05).

#### 3.2.2. Effects on the Embryonic Heart Rate

Heart rate was measured in zebrafish embryos at 48 hpf. Compared with the CK group, all the exposure groups presented a decrease in heart rate (Figure 3B). The greatest reduction was observed in the 100 µg/L SMX group, followed by the Ps + 100 µg/L SMX coexposure group. However, none of these changes reached statistical significance (*p* > 0.05).

#### 3.2.3. Effects on the Growth of Zebrafish Larvae

**Figure 4:**
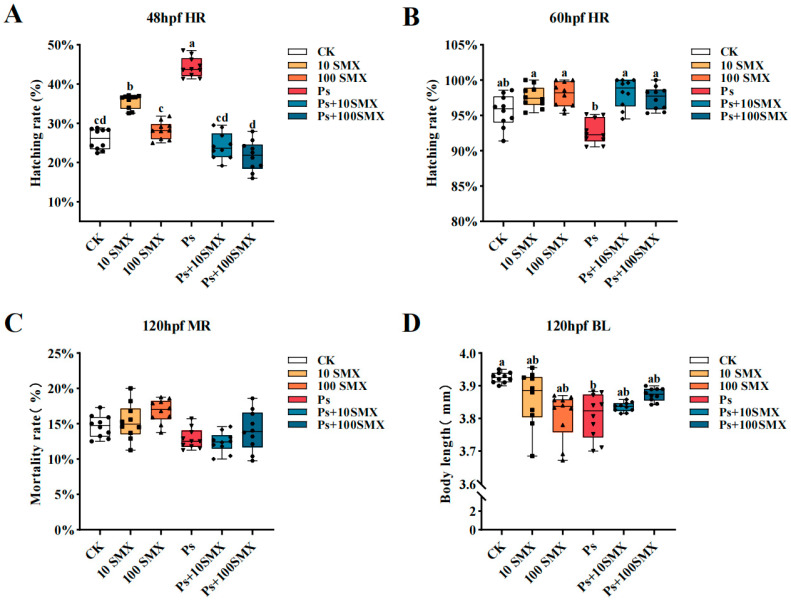
Effects of PS-MPs and SMX exposure on the growth and development of embryo–larval zebrafish. (**A**) Hatching rate at 48 hpf, (**B**) Hatching rate at 60 hpf, (**C**) Cumulative mortality at 120 hpf, (**D**) Body length at 120 hpf. Note: *n* = 10 replicates (15 larvae each), totaling 150 larvae per group (Different lowercase letters refer to differences at significance level *p* < 0.05 among different exposure groups).

At 48 hpf, compared with the CK group, both the 10 µg/L SMX group and the PS-MPs group presented significantly greater hatching rates (*p* < 0.05; Figure 4A). However, as the SMX concentration increased to 100 µg/L, the hatching rate in the single-exposure group returned to a level comparable to that in the CK group. Coexposure significantly reduced the increase in hatching rates induced by single exposures (*p* < 0.05), and the hatching rate decreased with increasing SMX concentration. By 60 hpf, hatching was essentially complete in all groups except the PS-MPs group, and the hatching rates in these groups were greater than those in the CK group, although the differences were not statistically significant (*p* > 0.05; Figure 4B). Notably, although the PS-MPs group had the highest hatching rate (44.3%) at 48 hpf, this group had the lowest hatching rate among all exposure groups at 60 hpf (*p* < 0.05). The mortality at 120 hpf is presented in Figure 4C. Mortality increased with increasing SMX concentration in both the SMX treatment group and the coexposure group, but the difference was not statistically significant compared with that in the CK group (*p* > 0.05). At 120 hpf, the body length was shorter in all the exposure groups than in the CK group (Figure 4D), and the reduction was most pronounced in the PS-MPs group (*p* < 0.05). Under SMX exposure, body length decreased with increasing concentration, although the changes were not significant (*p* > 0.05).

#### 3.2.4. Effects on the Malformation Rate in Zebrafish Larvae

**Figure 5:**
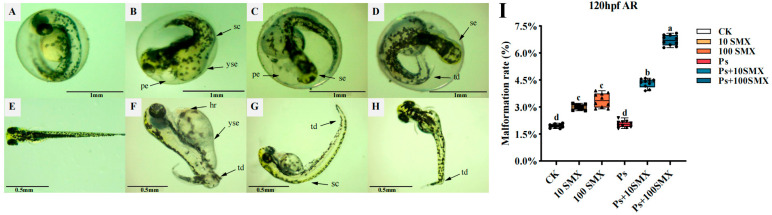
Typical malformations induced by individual or combined exposure to PS-MPs and SMX in embryo–larval zebrafish. (**A**,**E**) Control group, (**B**–**D**,**F**–**H**) Malformed groups. (**I**) Cumulative malformation rate at 120 hpf. hr, hemorrhage; pe, pericardial effusion; sc, spinal curvature; se, microphthalmia; td, tail deformity; yse, yolk sac edema. Note: *n* = 10 replicates (15 larvae each), totaling 150 larvae per group (Different lowercase letters refer to differences at significance level *p* < 0.05 among different exposure groups).

Exposure to PS-MPs or SMX, either individually or in combination, induced various malformations in larval zebrafish, with coexposure leading to more severe and morphological defects (Figure 5). The malformation rate was higher in coexposed larvae than in those exposed singly (Figure 5A–H). The observed malformations included hemorrhage, pericardial effusion and spinal curvature, microcephaly and microphthalmia, tail deformity, and yolk sac edema. No malformations were detected at 24 hpf. As exposure continued, by 48 hpf, the 10 µg/L SMX group exhibited three concurrent malformations, including pericardial effusion, yolk sac oedema, and spinal curvature. Similarly, the Ps + 10 µg/L SMX coexposure group showed not only pericardial effusion but also severe microcephaly and microphthalmia (Figure 5C,D).

With prolonged exposure, the malformations in larvae induced by PS-MPs or SMX could be categorized into two types: single malformations (pericardial effusion, yolk sac edema and tail deformity; Figure 5H) and complex malformations (spinal curvature, tail deformity combined with pericardial effusion; Figure 5G). Moreover, the frequency of these malformation symptoms further increased under coexposure, with some individuals even displaying four concurrent malformations (Figure 5F). Notably, a specific malformation, hemorrhage, was observed in larvae only under coexposure.

The malformation rate at 120 hpf is shown in Figure 5I, with significant differences among the treatment groups. Compared with that in the CK group, the malformation rate in the PS-MPs group did not significantly change, whereas compared with those in the CK group, the malformation rates in all the other treatment groups were significantly greater (*p* < 0.05).

Compared with that in the 10 µg/L SMX group, the malformation rate did not differ significantly in the 100 µg/L SMX group. However, compared with the CK group, the Ps + 100 µg/L SMX group presented a highly significant increase in the malformation rate (*p* < 0.01), which was also significantly greater than that of both the 100 µg/L SMX group and the PS-MPs group (*p* < 0.05). In summary, a high concentration of SMX (100 µg/L) significantly increased the malformation rate, and this pro-malformation effect was further enhanced when SMX was combined with PS-MPs.

### 3.3. Effects of PS-MPs and SMX on Neurodevelopment in Embryo–Larval Zebrafish

#### 3.3.1. Effects on the Contents of DA, 5-HT, and GABA

**Figure 6:**
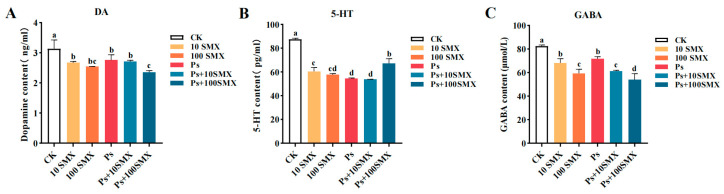
Effects of PS-MPs and SMX exposure on the content of DA (**A**), 5-HT (**B**) and GABA (**C**) in embryo–larval zebrafish (Note: Different lowercase letters refer to differences at significance level *p* < 0.05 among different exposure groups).

The different treatments significantly altered the DA content in the embryo–larval zebrafish (Figure 6A). Compared with the other treatment groups, the CK group presented significantly higher DA levels (*p* < 0.05). No significant differences were detected among the 10 µg/L SMX, PS-MPs, and Ps + 10 µg/L SMX treatments. However, the DA content was significantly greater in all three groups than in the Ps + 100 µg/L SMX group (*p* < 0.05). Although the DA concentration in the 100 µg/L SMX treatment group was greater than that in the Ps + 100 µg/L SMX treatment group, the difference was not statistically significant (*p* > 0.05). Both individual and combined exposure to SMX and PS-MPs significantly affected the DA content, with the most pronounced reduction occurring under high-concentration SMX combined with PS-MPs.

The 5-HT content was also significantly altered (Figure 6B). Compared with all the treatment groups, the control group presented significantly higher 5-HT levels (*p* < 0.05). The 5-HT content was significantly greater in the 10 µg/L SMX group than in the PS-MPs and Ps + 10 µg/L SMX groups (*p* < 0.05), although it remained lower than that in the control group. Notably, compared with the 100 µg/L SMX, PS-MPs, and Ps + 10 µg/L SMX groups, the Ps + 100 µg/L SMX group presented significantly higher 5-HT levels (*p* < 0.05). All treatments significantly altered the 5-HT content compared to the control, and compared with the other treatments, the combination of high-concentration SMX and PS-MPs resulted in a distinct response pattern.

As shown in Figure 6C, the content of GABA followed a trend similar to that of DA and 5-HT, with all treatment groups showing significantly lower levels than the CK group did (*p* < 0.05). The GABA content in the 10 µg/L SMX and PS-MPs groups was significantly greater than that in the 100 µg/L SMX, Ps + 10 µg/L SMX, and Ps + 100 µg/L SMX groups (*p* < 0.05). Furthermore, among all the treatments, the Ps + 100 µg/L SMX treatment resulted in the lowest GABA content (*p* < 0.05). The exposure to PS-MPs and SMX significantly affected GABA levels, with the most marked reduction again observed under high-concentration SMX combined with PS-MPs.

#### 3.3.2. Effects on the ACh Content and AChE Activity

**Figure 7:**
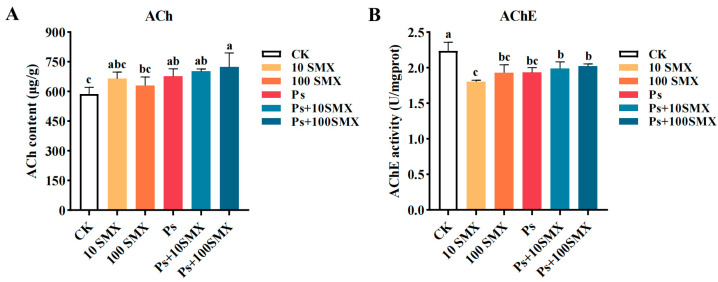
Effects of PS-MPs and SMX exposure on ACh content (**A**) and AChE activity (**B**) in embryo–larval zebrafish (Note: Different lowercase letters refer to differences at significance level *p* < 0.05 among different exposure groups).

PS-MPs and SMX modulated the acetylcholine (ACh) content in the embryo–larval zebrafish (Figure 7A). Compared with those in the control group, the ACh levels in the treatment groups increased significantly after individual or combined exposure (*p* < 0.05). The Ps + 100 µg/L SMX group presented the highest ACh content, which was significantly greater than that in the 100 µg/L SMX group (*p* < 0.05). Notably, no significant differences in the ACh content were detected among the 10 µg/L SMX, 100 µg/L SMX, PS-MPs, and Ps + 10 µg/L SMX groups (*p* > 0.05). In summary, both individual and combined exposures effectively elevated ACh levels, with a synergistic effect at high SMX concentrations leading to a pronounced increase.

PS-MPs and SMX also significantly suppressed AChE activity (*p* < 0.05; Figure 7B). The lowest AChE activity was recorded in the 10 µg/L SMX group, whose activity was significantly lower than that in the Ps + 10 µg/L SMX group (*p* < 0.05). Moreover, no statistically significant differences in AChE activity were detected among the 100 µg/L SMX, PS-MPs alone, Ps + 10 µg/L SMX, and Ps + 100 µg/L SMX groups (*p* > 0.05). These results indicate that both individual and combined treatments significantly inhibited AChE activity, with the most severe inhibitory effect observed under 10 µg/L SMX exposure.

#### 3.3.3. Effects on Neural Gene Expression in Embryo–Larval Zebrafish

**Figure 8:**
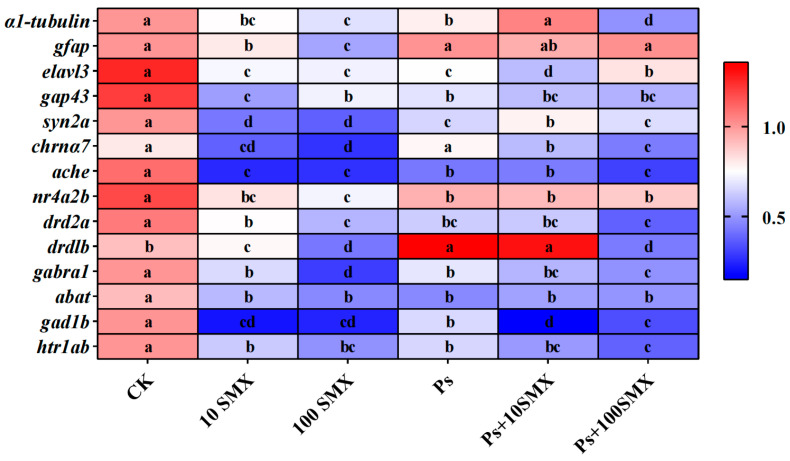
Effects of PS-MPs and SMX exposure on the expression of neurodevelopment- and neurotransmission-related genes in embryo–larval zebrafish (Note: Different lowercase letters refer to differences at significance level *p* < 0.05 among different exposure groups).

These results indicate that PS-MPs and SMX induce significant alterations in neurotransmitter activity. To further explore their roles in neurotoxicity, the relative expression of genes associated with early neurodevelopment (*α1-tubulin*, *gfap*, *elavl3*, *gap43*, *syn2a*), acetylcholine receptors (*chrna7*), acetylcholinesterase (*ache*), DA signalling (*nr4a2b*, *drd2a*, *drd1b*), GABA signalling (*gabra1*, *abat*, *gad1b*), and serotonin signalling (*hrlαb*) was measured by quantitative real-time PCR (Figure 8).

The expression of *α1-tubulin* was altered in all treatment groups, with significant downregulation in the Ps + 100 µg/L SMX group (*p* < 0.05). *gfap* expression significantly decreased in the 100 µg/L SMX group (*p* < 0.05). The expression of the neurodevelopmental genes *elavl3*, *gap43*, and *syn2a* decreased across all treatments (*p* < 0.05). Among them, *elavl3* showed the most pronounced downregulation in the Ps + 10 µg/L SMX group. However, compared with that in the PS-MPs, 100 µg/L SMX, and Ps + 10 µg/L SMX groups, the expression of *elavl3* was significantly upregulated in the Ps + 100 µg/L SMX group (*p* < 0.05). The expression of *syn2a* was significantly lower in the 10 µg/L SMX and 100 µg/L SMX groups (*p* < 0.05), although it recovered in the Ps + 10 µg/L SMX and Ps + 100 µg/L SMX groups and remained below the CK level. With respect to *chrna7*, PS-MPs did not affect its expression, whereas the expression in both the SMX and coexposure groups decreased in a concentration-dependent manner with increasing SMX. The *ache* gene was significantly downregulated in all treatment groups compared with the CK group (*p* < 0.05). The expression of *nr4a2b* did not differ significantly between the 10 µg/L SMX and 100 µg/L SMX groups but was upregulated to varying degrees in the PS-MPs, Ps + 10 µg/L SMX, and Ps + 100 µg/L SMX groups. In contrast, *drd2a* was significantly downregulated in the groups treated with 10 µg/L SMX, 100 µg/L SMX, Ps + 10 µg/L SMX, and Ps + 100 µg/L SMX, with the reduction becoming more pronounced as the SMX concentration increased (*p* < 0.05). The expression of the *drd1b* gene was significantly upregulated in the PS-MPs and Ps + 10 µg/L SMX groups (*p* < 0.05) but downregulated in the 10 µg/L SMX, 100 µg/L SMX, and Ps + 100 µg/L SMX groups (*p* < 0.05). The expression levels of *gabra1*, *abat*, *gad1b*, and *hrlαb* were significantly lower in all the exposure groups than in the CK group (*p* < 0.05). With respect to *gabra1*, *abat*, and *hrlαb*, the expression decreased with increasing SMX concentration in both the SMX and coexposure groups, with some groups showing statistically significant differences (*p* < 0.05). The expression of *gad1b* was significantly downregulated in the 10 µg/L SMX, 100 µg/L SMX, Ps + 10 µg/L SMX, and Ps + 100 µg/L SMX groups, whereas it was significantly upregulated in the PS-MPs group (*p* < 0.05).

### 3.4. Integrated Biomarker Response (IBR) Index

**Figure 9:**
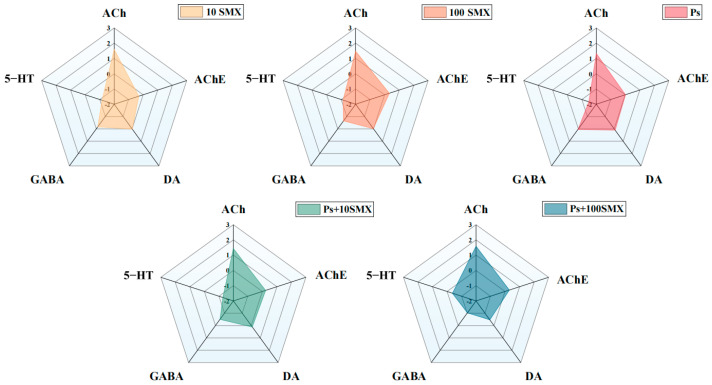
Radar plot of the IBR index for embryo–larval zebrafish exposed to PS-MPs and SMX.

**Figure 10:**
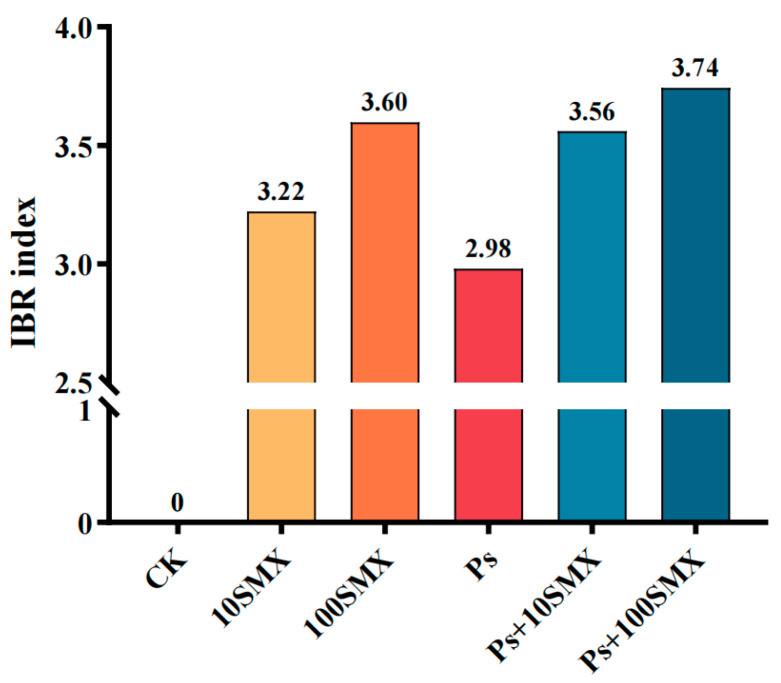
IBR calculated from biochemical parameters after 120 h exposure to PS-MPs and SMX.

On the basis of the change in the levels of five biomarkers, DA, 5-HT, GABA, ACh, and AChE, the IBR index was calculated after 120 h of exposure. As shown in Figure 9, the points on each axis represent the standardized values of the indicators (values > 0 indicate induction, and values < 0 indicate suppression), allowing comparison of toxic effect profiles across different exposure groups. The results revealed that the ACh content was the most sensitive indicator of toxicity. The IBR indices for the SMX treatment groups were 3.22 and 3.60, respectively, demonstrating a concentration-dependent toxic effect. In contrast, the IBR index for the coexposure groups increased to 3.56 and 3.74, which were higher than those of the corresponding SMX group, indicating a synergistic increase in SMX toxicity by PS-MPs. The IBR calculated as the sum of the absolute deviation coefficients for the five biomarkers, is shown for each treatment after 120 h of exposure (Figure 10). The results indicate that coexposure groups exhibited higher IBR than the corresponding single-exposure groups, demonstrating enhanced combined toxicity of PS-MPs and SMX at the tested concentrations.

## 4. Discussion

### 4.1. Distribution of PS-MPs in Embryo–Larval Zebrafish

MPs have been widely detected in various water environments globally [24,25]. As key components of aquatic ecosystems, organisms can accumulate MPs through direct ingestion, leading to toxic effects. In this study, the distribution of 1 µm PS-MPs was investigated using an embryo–larval zebrafish model. PS-MPs adhered only to the chorion surface and did not penetrate into the embryo during prehatching. This may be attributed to the physical barrier formed by the chorion, as the diameter of its pores (approximately 0.5–0.7 µm) is smaller than the size of the PS-MPs [26]. Notably, even smaller nanoplastics (100 nm) can be effectively blocked by the chorion [27], indicating a general barrier function of this structure against fine particles.

After continuous exposure for 60 h, the fluorescence intensity in larval zebrafish decreased significantly. This reduction may be because most embryos had completed hatching by this time, detaching from the microplastic-chorion, while the newly hatched larval zebrafish had not yet been substantially contaminated by MPs. Compared with those in the embryonic stage, the uptake and accumulation of microplastics in zebrafish larvae are more complex. At 72 h post-hatching, PS-MPs were first detected in the intestine, and by 108 h, apparent accumulation was also observed in the gills, with accumulation levels increasing. This distribution pattern aligns with previous reports that ingested PS-MPs primarily accumulate in the digestive system [28,29]. The 1 µm PS-MPs were retained mainly in the intestine, with no significant signal detected in the brain or liver, suggesting that smaller particles tend to accumulate in the gut [30]. Similarly, other studies have shown that microplastics often remain in the digestive tract and do not readily enter internal tissues [31]. Although some studies have reported that small-sized MPs (e.g., <180 nm) can enter the brain in early development [32], and particles < 500 nm can efficiently cross the gastrointestinal barrier and accumulate in the liver [33]. The absence of 1 µm PS-MPs in other organs in this study may be due to their larger size hindering penetration of biological barriers or possibly to partial degradation of the particles within the intestine [33,34].

### 4.2. Developmental Toxicity of PS-MPs and SMX in Embryo–Larval Zebrafish

Developmental toxicity is a key indicator for assessing the impact of environmental pollutants on embryo–larval zebrafish [35]. Spontaneous movement, which reflects the initial functional state of the neuromuscular system, is highly sensitive to pollutant exposure [36]. The results indicate that the combined exposure group (Ps + 10 µg/L SMX) exhibited significantly higher spontaneous movement counts compared to the single 10 µg/L SMX exposure group. Conversely, while the movement counts in the combined exposure group were also numerically higher than those in the Ps group, this difference did not reach statistical significance. Wang et al. [5] exposed zebrafish embryos to polystyrene nanoplastics and polybrominated diphenyl ethers, finding that coexposure increased the spontaneous movement of the embryos compared to exposure to nanoplastics alone. Interestingly, in the present study, coexposure induced a nonsignificant increase in spontaneous movement, which could represent a low-dose excitatory effect or a compensatory response to stress.

The cardiovascular system is another key target of pollutant toxicity, with heart rate serving as a core indicator of circulatory function [37]. The heart rate tended to decrease in all the exposure groups at 48 hpf, most notably in the 100 µg/L SMX group. A reduced heart rate is typically accompanied by decreased circulatory and metabolic efficiency, providing a potential physiological basis for the subsequently observed pericardial effusion and growth inhibition. Both 10 µg/L SMX and PS-MPs exposure significantly promoted hatching at 48 hpf. However, when the SMX concentration was increased to 100 µg/L or combined with PS-MPs, this effect disappeared, and the hatching rates returned to or fell below control levels, which is consistent with previous reports [38]. Notably, while the highest hatching rate occurred in the PS-MPs group at 48 hpf, this group exhibited the lowest hatching rate at 60 hpf. This pattern suggests that PS-MPs may initially stimulate chorion rupture but subsequently delay the hatching of the remaining embryos, possibly by impairing gas exchange or nutrient absorption during prolonged exposure.

Body length is a comprehensive indicator reflecting overall nutritional status, protein synthesis efficiency, and energy metabolism [39]. At 120 hpf, body length was reduced in all the exposure groups relative to that in the control group, with the most significant shortening observed in the PS-MPs group, indicating a sustained inhibitory effect of PS-MPs on larval growth. This growth retardation may be closely related to the aforementioned physiological disturbances, such as decreased heart rate and weakened metabolism.

Morphological development is the integrated phenotypic output of precisely regulated embryonic processes and serves as an important endpoint for evaluating pollutant toxicity in embryo–larval zebrafish [35]. Both individual and combined exposure to SMX and PS-MPs induced various malformations, with coexposure resulting in significant synergistic toxicity. At the initial stage of exposure, SMX could induce pericardial effusion and yolk sac edema, indicating its potential interference with cardiovascular function and energy metabolism. Cardiac toxicity in zebrafish is characterized by pericardial effusion and bradycardia [40,41]. With prolonged exposure, the synergistic teratogenic effect of coexposure became more pronounced. While individual exposures typically resulted in one or two concurrent malformations, the coexposure groups frequently exhibited three or even four malformations, accompanied by hemorrhage. This complex malformation pattern resembles findings from studies on other pollutant mixtures, such as nanoplastics and polybrominated diphenyl ethers [5]. Quantitative analysis further revealed that compared with the control treatment, the combination of PS-MPs with SMX did not significantly increase the malformation rate, leading to a marked increase in malformations. These findings suggest that PS-MPs can act as environmental stressors or pollutant carriers to significantly increase the toxicity of SMX [14].

### 4.3. Effects of PS-MPs and SMX on Neurotransmitter Activity and Enzyme Activity in Embryo–Larval Zebrafish

Disruption of neurotransmitter homeostasis is a core mechanism through which exogenous pollutants induce developmental neurotoxicity and behavioural abnormalities [42]. DA, 5-HT, and GABA are key neurotransmitters that regulate movement, mood, and neuronal survival, and changes in their levels directly reflect the intensity and characteristics of neurotoxicity [43]. Both individual and combined exposure to SMX and PS-MPs reduced the DA and GABA levels. Previous studies have shown that decreased DA leads to motor inhibition [43], while reduced GABA may exacerbate anxiety and motor abnormalities [44]. Therefore, the decrease in DA and GABA induced by coexposure in this study may be important contributors to the observed behavioural disturbances in larvae. Furthermore, disruption of DA and GABA homeostasis can trigger caspase activation and apoptosis [45], representing a potential mechanism of neuronal damage. In contrast, the response pattern of 5-HT was more complex, with its content increasing in the coexposure group. This response may be attributed to the context-dependent actions of 5-HT mediated by distinct receptors and targets [46]. A full understanding requires further investigation integrating oxidative stress markers and transcriptional profiling [47].

ACh, a key neurotransmitter in the cholinergic system, is involved in motor control, neuromuscular signalling, and cognitive regulation. AChE, the rate-limiting enzyme for ACh metabolism, maintains synaptic homeostasis by catalyzing ACh hydrolysis, and its abnormal activity is a classic biomarker of neurotoxicity [48,49]. The widespread inhibition of AChE activity observed in this study led to abnormal accumulation of synaptic ACh, which is consistent with reports following exposure to various environmental toxicants [49,50,51]. As a dual-effect neurotransmitter with both excitatory and inhibitory properties [52], the abnormal accumulation of ACh may have complex regulatory effects on neural circuits depending on the specific targets involved. In this study, only total ACh content and AChE activity were measured. Future work should integrate indicators such as choline acetyltransferase (ChAT) activity and cholinergic receptor expression levels to further elucidate the molecular regulatory mechanisms underlying the effects of SMX and PS-MPs coexposure on the cholinergic system.

### 4.4. Effects of PS-MPs and SMX on Neural Gene Expression in Embryo–Larval Zebrafish

The stable expression of genes related to neurodevelopment and neurotransmitter pathways is the molecular basis for maintaining normal development and function of the nervous system in embryo–larval zebrafish [53,54]. PS-MPs and SMX exposure broadly disrupted the gene expression profile of the zebrafish nervous system, affecting genes involved in neurodevelopment and the cholinergic, dopaminergic, GABAergic and serotonergic systems, with clear dose- and combination-dependent effects. The widespread downregulation of the core neurodevelopmental genes *elavl3*, *gap43*, and *syn2a* points to impairments in neuronal differentiation, axonal growth, and synapse formation, respectively, aligning with the neurotoxic mechanisms of various pollutants [53,55,56,57,58]. These findings are highly consistent with reports that pollutants such as difenoconazole [57] and sulfoxaflor [58] downregulate the expression of similar genes, thereby damaging neuronal differentiation, axonal growth, and synaptogenesis. Furthermore, significant suppression of *α1-tubulin* (an essential microtubule cytoskeleton gene) under coexposure may disrupt neuronal structural integrity, similar to the mechanism reported for microcystin-LR toxicity [59,60]. In contrast, the limited response of *gfap* (an astrocyte marker), which was downregulated only in the high-concentration SMX group, may be due to the relatively large size of the PS-MPs used in this study hindering their ability to cross the blood–brain barrier and directly affect glial cells. These results contrast with those of studies in which sulfoxaflor exposure led to widespread downregulation of *gfap* [58].

Changes in cholinergic system-related gene expression revealed interactions between PS-MPs and SMX. The downregulation of *ache*, the gene encoding AChE, directly led to inhibited AChE activity and ACh accumulation [20,61], which is consistent with findings from multiple microplastic exposure studies in clams [62] and Nile tilapia [63]. The concentration-dependent downregulation of *chrna7* with increasing SMX may represent a compensatory reduction in receptor expression to counterbalance the excitatory effects of ACh accumulation, a finding that complements previous research focused primarily on the degradation pathway. Notably, PS-MPs alone did not significantly affect *chrna7* expression but did increase ACh levels, indicating that PS-MPs mainly regulate ACh homeostasis by inhibiting ache or AChE activity. In the GABA system, PS-MPs may upregulate *gad1b* as a compensatory mechanism, whereas the addition of SMX suppresses this gene, ultimately impairing GABA synthesis. The upregulation of *nr4a2b* (critical for dopaminergic progenitor maturation and differentiation [64]) under PS-MPs exposure, despite decreased DA levels, may reflect an adaptive response by the nervous system to promote dopaminergic neuron differentiation and compensate for DA deficiency. The synergistic downregulation of the dopamine receptor gene *drd2a* with reduced DA levels disrupts signal transduction and feedback regulation [65]. Decreases in the expression of *gabra1* (which encodes the GABA receptor) with increasing SMX concentration impair GABA receptor function. The upregulation of the key GABA synthesis gene *gad1b* in the PS-MPs group and its downregulation in the coexposure groups explain the mild reduction in GABA expression in response to PS-MPs alone and the marked decrease in expression in response to coexposure. The development of astrocytes in zebrafish embryos depends on the regulation of thyroid hormones (T3/T4) [66], and SMX may interfere with the synthesis of thyroid hormones or their binding to receptors. Impairment of the thyroid hormone signalling pathway can prevent astrocytes from maturing properly, leading to the downregulation of *gfap* gene expression. In contrast, PS-MPs do not interfere with these pathways; their toxicity may stem from mechanical damage or non-specific inflammation, which is unrelated to the regulatory mechanisms of the *gfap* gene. These targeted gene disturbances, combined with the widespread suppression of developmental genes such as *elavl3*, *gap43*, and *syn2a*, underlie the comprehensive disruption of the neurotransmitter system.

### 4.5. Integrated Biomarker Response Index

The IBR index is an effective quantitative tool for assessing the combined stress effects of pollutants. By integrating multilevel biomarker responses, the IBR visually reflects the overall health impairment of organisms, with its value positively correlated with stress intensity [67,68,69]. After 120 h of individual exposure, the IBR value for SMX was greater than that for PS-MPs in embryo–larval zebrafish. In the coexposure groups, the IBR value for Ps + 100 µg/L SMX exceeded that of the corresponding individual groups, indicating that PS-MPs exacerbated the neurotoxicity of SMX. Yu et al. [70] similarly reported a higher IBR index for combined exposure to microplastics and oxytetracycline in zebrafish than for individual exposures, indicating greater combined toxicity. In summary, together with the significant changes in neurotransmitter activity and enzyme activity, the IBR results confirm that PS-MPs and SMX exert multifaceted neurotoxic effects on embryo–larval zebrafish and exhibit clear synergistic interactions.

## 5. Conclusions

This study demonstrated that PS-MPs and SMX accumulate primarily in the intestine and gills of embryo–larval zebrafish, triggering multilevel toxic effects. At the physiological developmental level, individual exposure reduced heart rate and increased hatchability, malformation rate, and spontaneous movement abnormalities while significantly inhibiting body length. Compared with single exposures, coexposure resulted in synergistic increases in spontaneous movement frequency and malformation rate. Neurochemical analyses revealed that both single and combined exposures inhibited acetylcholinesterase activity, disrupted monoamine neurotransmitter homeostasis (reducing DA, GABA, and 5-HT levels), and caused cholinergic system dysfunction (elevating the ACh content). Furthermore, analysis of neural gene expression indicated that coexposure downregulated key neurodevelopmental genes (*gap43* and *elavl3*), disrupted cholinergic system genes (*chrna7* and *ache*), and reduced the expression of genes involved in the dopamine signalling pathway (*nr4a2b*). In summary, the combined effects of PS-MPs and SMX induce significant developmental and neurotoxicity through synergistic interference with neurotransmitter levels and gene expression. At the physiological developmental level, individual exposure reduced heart rate and increased hatchability, malformation rate, while significantly inhibiting body length.

## Figures and Tables

**Table 1:** Primers’ information.

Gene Name	Sequence of the Primer (5′-3′)	Annealing Temperature (°C)	Accession Number
*β-actin*	F:ACAGGGAAAAGATGACACAGATCA R:CAGCCTGGATGGCAACGTA	60.08	NM_181601.5
*α1-tubulin*	F:AATCACCAATGCTTGCTTCGAGCC R:TTCACGTCTTTGGGTACCACGTCA	59.92	NM_194388.2
*gfap*	F:GGATGCAGCCAATCGTAAT R:TTCCAGGTCACAGGTCAG	55.74	NM_131373.2
*elavl3*	F:AGACAAGATCACAGGCCAGAGCTT R:TGGTCTGCAGTTTGAGACCGTTGA	62.57	NM_131449.1
*gap43*	F:TGCTGCATCAGAAGAACTAA R:CCTCCGGTTTGATTCCATC	55.66	NM_131341.2
*syn2a*	F:GATCATGTAACGTGGCTGTGC R:ATCAGACATGCAAATGCCCA	59.94	NM_001002597.2
*chrnα7*	F:GAGTGGGACCTTGTGGAAGT R:TCCGCATCACCACCGTAAAA	59.96	NM_201219.2
*ache*	F:CTCCAGGAACACTAGGCTGG R:AGGTACACAGCACCATGCGA	59.46	NM_131846.2
*nr4a2b*	F:AGCTCCTTTTCGAGTCTGCC R:CTCTGGCAGGTTGGATCTGTA	60.04	NM_001002406.1
*drd2a*	F:TGGTACTCCGGAAAAGACG R:ATCGGGATGGGTGCATTTC	57.88	NM_183068.1
*drdlb*	F:TTCTCCATCTTGCTGGGCTG R:AGGGCCACAGTTGTTGTTGA	60.03	NM_001135976.2
*gabra1*	F:TGCGATACATTCCGGGTGAG R:AGGATGGCTCGAACGTTTCC	60.39	NM_001077326.1
*abat*	F:GTCCGACTGTGCTCCTCACT R:CCTTCGTGTCTGACGTTCGAG	61.53	NM_201498.2
*gad1b*	F:TCGTGTGTATCTCTGACTGAATG R:TGCAACAATCGCCTGAGTTG	59.41	NM_194419.1
*htr1ab*	F:GCTCGGCTATTCAAACTCGC R:ACGCGCTCTGGAAGTCTTTA	59.70	NM_001145766.1

## Data Availability

The original contributions presented in the study are included in the article, further inquiries can be directed to the corresponding authors.

## Supplementary Materials

The following supporting information can be downloaded at: https://www.mdpi.com/article/10.3390/toxics14010074/s1, Figure S1: Characterization of PS-MPs. (A) Image of PS-MPs; (B) Image of red fluorescent PS-MPs; (C) Fluorescence microscopy image of red fluorescent PS-MPs.; Text S1. Neurotransmitter content; Table S1: Operation steps for AchE activity determination.
