# Supplementary material for: Toxic Effects of Polystyrene Microplastics and Sulfamethoxazole on Early Neurodevelopment in Embryo–Larval Zebrafish (Danio rerio)
Source: Toxics. 2026 Jan 14;14(1):74. doi: 10.3390/toxics14010074 (PMC12845721; doi:10.3390/toxics14010074)
Supplement: Supplementary file 1 [file toxics-14-00074-s001.zip › toxics-4078319-supplementary.pdf]

Toxic effects of polystyrene microplastics and sulfamethoxazole on early neurodevelopment in embryo–larval zebrafish (*Danio rerio*)

Fantao Meng , Shibo Ma , Yajun Wang , Chunmei Wang , Ruoming Li* and Jiting Wang*

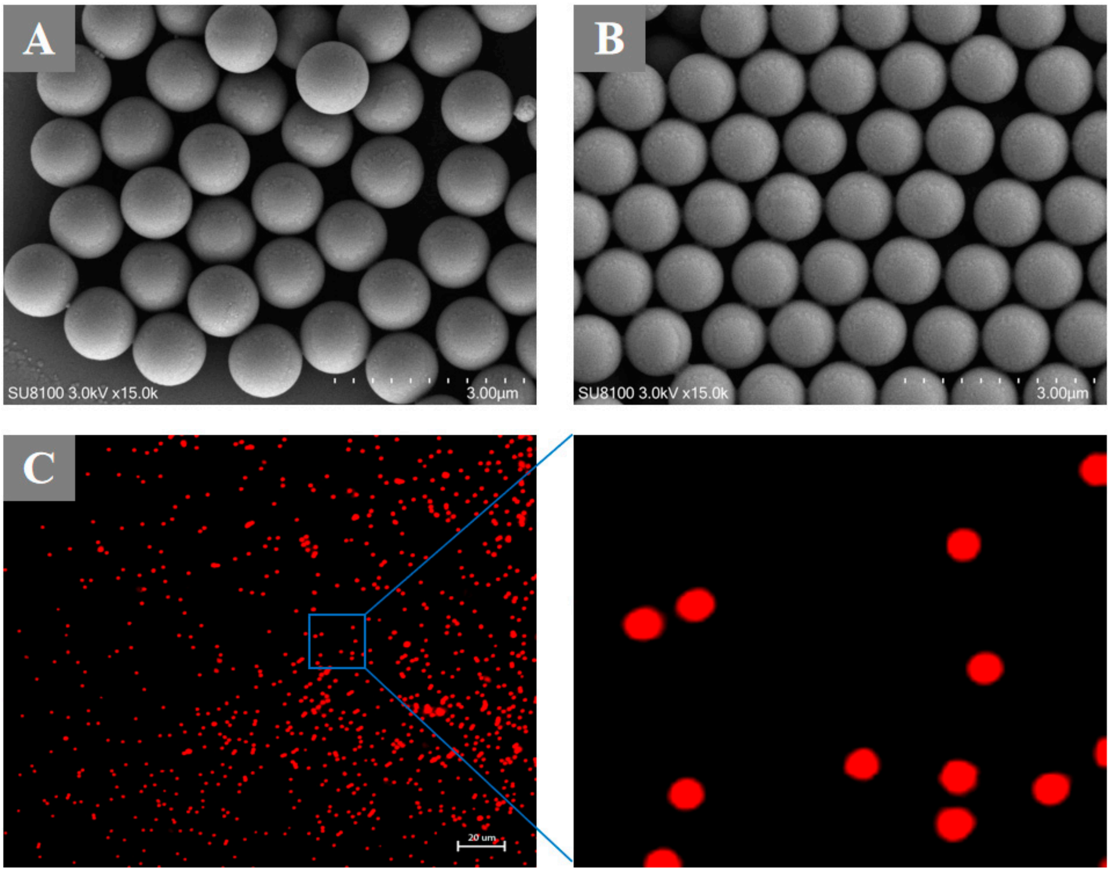

Figure S1. Characterization of PS-MPs. (A) Image of PS-MPs; (B) Image of red fluorescent PS-MPs; (C) Fluorescence microscopy image of red fluorescent PS-MPs.

Text S1. Neurotransmitter content

The detections of DA, 5-HT, and GABA contents in zebrafish

Larval tissues were accurately weighed and homogenized on ice in normal saline at a tissue-to-solution ratio of 1:9 (w/v). The homogenate was centrifuged at 4 °C and 3000 rpm for 20 min. For the ELISA, 10 μL of supernatant was added to the sample wells, followed by 40 μL of sample dilution buffer. Standard wells received 50 μL of serially diluted standards. Then, 100 μL of horseradish peroxidase (HRP)-conjugated detection antibody was added to all standard and sample wells. After sealing, the plate was incubated at 37 °C for 1 h. The liquid was discarded, and each well was washed five times with wash buffer, followed by blotting on

absorbent paper. Subsequently, 50 μL each of substrate A and B were added, and the plate was incubated at 37 °C in the dark for 10 min for color development. The reaction was stopped by adding 50 μL of stop solution. Absorbance (OD) was measured at 450 nm using a microplate reader, and sample concentrations were calculated based on the standard curve.

The detection of ACh content in zebrafish

Larval tissues were accurately weighed and homogenized in extraction buffer at a tissue-to-buffer ratio of 1:9 (w/v). The homogenate was centrifuged at 3500 rpm for 10 min, and the supernatant was collected. For acetylcholine quantification, 25 μL of supernatant was mixed with the working solution and incubated at room temperature for 15 min. Subsequently, 50 μL of stop reagent, 50 μL of chromogenic reagent, and 25 μL of clarifying reagent were added sequentially, followed by mixing and a further 10 min incubation. Finally, 200 μL of the reaction mixture was transferred to a 96-well plate, and the absorbance of each well was measured at 550 nm using a microplate reader.

The detections of TP content and AChE activity in zebrafish

Sample preparation and analytical procedures were as follows: Larval tissues were accurately weighed and homogenized in extraction buffer at a tissue-to-buffer ratio of 1:9 (w/v). The homogenate was centrifuged at 5000 rpm for 10 min, and the supernatant was collected. The microplate reader was pre-warmed for 20 minutes prior to measurement. The specific operating procedure is detailed in Table S1.

Table S1. Operation steps for AchE activity determination.

Tube Type	Test Tube	Control Tube	Standard Tube	Blank Tube
Sample (μL)	15			
1 μmol/mL Standard Working Solution (μL)			15	
ddH$_2$O (μL)				15
Substrate Buffer (mL)	0.5	0.5	0.5	0.5
Chromogenic Working Solution (mL)	0.5	0.5	0.5	0.5
Mix well and incubate at 37 °C for 6 min.				
Inhibitor (mL)	0.03	0.03	0.03	0.03
Clearing Agent (mL)	0.1	0.1	0.1	0.1
Test（μL）		15		
Mix thoroughly, allow to stand at room temperature for 15 min, and then measure the absorbance (OD) of each tube at 412 nm with a 0.5 cm light path, using double-distilled water as blank.				

Protein concentration was determined using the BCA method according to the following procedure: Larval tissues were accurately weighed and homogenized on ice in normal saline

at a tissue-to-saline ratio of 1:9 (w/v). The homogenate was centrifuged at 2500 rpm for 10 min. The supernatant was collected and diluted 1:9 with normal saline to obtain a 1% tissue homogenate. A standard solution with a concentration of 524 μg/mL was prepared. Then, 10 μL of distilled water, standard solution, or test sample was added to blank, standard, and test wells, respectively. 250 μL of working solution was added to each well, the plate was gently shaken to mix, and incubated at 37 °C for 30 min. Absorbance (OD) was measured at 562 nm using a microplate reader. AChE activity (U/mg) was calculated based on the measured OD value and the protein concentration of the sample.
